# Items, News and Gossip

**Published:** 1872-07

**Authors:** 


### Items, News and Gossip.

Several St. Louis physicians commend in cerebro-spinal meningitis, counter-irritation, scarifying and cupping along the spine, free purgation by calomel and jalap, followed by large doses of quinina. All members of the local society present concurred in this treatment; but it was remarked by one gentleman (Dr. Gill), that "he had never seen a case recover when the petechia was well marked." —— The "thirst cure," which Dr. Watson commends in acute catarrh or "bad cold," is reported by Dr. Pimser, of Germany, as very favorable in result in pleurisy, very certain and rapid. The plan is to commence deprivation of all fluids immediately on cessation of the inflammatory stage. It is claimed that the exuded material is rapidly reabsorbed, the formation of fibrinous bands and false membrane prevented, and the lung completely relieved of compression.——M. Broca (*Lyon Medicale*), in the reduction of dislocations or fractures with displacement, writes that he secures relaxation of the muscles, without the use of anæsthetics, by compressing the principal artery supplying the limb. It being, at least, probable that in the majority of cases the resistance to replacement is not simply muscular, the importance and value of the discovery seems at present somewhat questionable.—— Surgeon Wood (*London Lancet*), advises in tracheotomy a transverse rather than a vertical cut, as being easier kept open, especially when the patient, as he usually does, inclines to throw the head backward. With a sharp small-bladed knife he makes a single transverse incision across the lower part of the hollow depression felt by the finger, just above the cricoid ring, through the skin and membrane at once, right into the windpipe. A tube is not generally necessary, but if used, should be modified to suit the opening from the one in ordinary use.——The Legislature of Kentucky has passed a bill subjecting habitual opium eaters to similar legal guardianship with drunkards and lunatics.——To render readily inflammable fabrics incombustible, a recent writer advises their immersion in a mixture of four parts of borax and three parts of sulphate of magnesia in from twenty to thirty parts of water. The solution is to be used immediately, or else the insoluble borate of magnesia is formed. For cheaper fabrics a solution of sulphate of ammonia and gypsum may be employed. The *Reporter* has the item.——Cundurango flickers once more, but this time rather as an anti-syphilitic than as an *anti-canceric.* Prof. ANDREWS publishes letters from Ecuador, that speak almost as highly of its uses as their predecessors did of Sarsaparilla. One of the tropical doctors offers to lay it down in New York at two dollars a pound. Poor Bliss & Keene have been asking *forty.* If it does not cure, the article is not genuine — (which remark we think we have seen in some newspaper advertisement). ——Benzine internally administered, ten to twenty drop doses, is one of the latest notions for hooping cough. It may also be used with the atomizer.——W. W. Dawson, M.D., President of the Ohio Med. Soc., in his annual address, after recapitulating the triumphs of *American* Medicine and Surgery, makes this pertinent inquiry: "If our system of medical education be so defective, why this great excellence in the men who have been educated by it? It is unusual to see such fruit from a tree that is all unsound." We, also, pause for a reply.——Dr. Harshberger (*Reporter*), gives a case where inflammation of the tongue, mouth and throat, of a violent character, with severe constitutional symptoms, was rapidly relieved by local application of ice, small lumps being kept constantly in the mouth, and the neck surrounded by a towel wet in ice water. The treatment was suggested by Dr. Corson's ice-treatment in scarlatina, measles, diphtheria, etc.—— The local application of a strong solution (85 per cent.) of carbolic acid, as an anæsthetic, is urged by Dr. Andrew H. Smith, (*N. Y. Medical Journal*). Under its application the cuticle speedily becomes whitened, shriveled and insensible, so that the contact of the knife is unfelt. This may be taken advantage of in opening whitlows, felons, boils, etc. The Dr. also recommends it as a painless revulsive, and also inhaled in the form of spray for relieving irritation of the bronchial mucous membrane.——Dr. Todd (*Ind. Jour.*) prefers the powdered calabar bean to other preparations. In cerebro-spinal meningitis he commences with four grains of the powder, and gives three grains every hour after until the urgent distress of the back part of the head and neck is relieved. In his experience this is usually secured in from three to five hours. The drug was not pushed far enough to produce contraction of the pupil, though some depression was complained of. Subsequently the medicine was continued at such intervals as the case required. After ten days, more or less, bromide of ammonium was substituted. From our own observation and such reports as we have read, we doubt any relation between the bean, in any shape, and the disease.

